# Grapiprant: an EP4 prostaglandin receptor antagonist and novel therapy for pain and inflammation

**DOI:** 10.1002/vms3.13

**Published:** 2015-12-21

**Authors:** Kristin Kirkby Shaw, Lesley C. Rausch‐Derra, Linda Rhodes

**Affiliations:** ^1^ Aratana Therapeutics, Inc. Kansas City Kansas USA; ^2^ Animal Surgical Clinic of Seattle Seattle Washington USA

**Keywords:** osteoarthritis, EP4, grapiprant, pain, piprant, prostaglandin

## Abstract

There are five active prostanoid metabolites of arachidonic acid (AA) that have widespread and varied physiologic functions throughout the body, including regulation of gastrointestinal mucosal blood flow, renal haemodynamics and primary haemostasis. Each prostanoid has at least one distinct receptor that mediates its action. Prostaglandin E_2_ (PGE_2_) is a prostanoid that serves important homeostatic functions, yet is also responsible for regulating pain and inflammation. PGE_2_ binds to four receptors, of which one, the EP4 receptor, is primarily responsible for the pain and inflammation associated with osteoarthritis (OA). The deleterious and pathologic actions of PGE_2_ are inhibited in varying degrees by steroids, aspirin and cyclo‐oxygenase inhibiting NSAIDs; however, administration of these drugs causes decreased production of PGE_2_, thereby decreasing or eliminating the homeostatic functions of the molecule. By inhibiting just the EP4 receptor, the homeostatic function of PGE_2_ is better maintained. This manuscript will introduce a new class of pharmaceuticals known as the piprant class. Piprants are prostaglandin receptor antagonists (PRA). This article will include basic physiology of AA, prostanoids and piprants, will review available evidence for the relevance of EP4 PRAs in rodent models of pain and inflammation, and will reference available data for an EP4 PRA in dogs and cats. Piprants are currently in development for veterinary patients and the purpose of this manuscript is to introduce veterinarians to the class of drugs, with emphasis on an EP4 PRA and its potential role in the control of pain and inflammation associated with OA in dogs and cats.

## Introduction

Prostanoids are metabolites of arachidonic acid (AA) and have been the target of medicinal therapies for centuries (Appelboom 2002). However, until recently, preparations aimed at inhibiting the action of prostanoids have, with varying degrees of prostanoid specificity, prevented their production and thus impacted both pathologic and homeostatic activity (Curry *et al*. 2005; KuKanich *et al*. 2012). The evolution of pain management has entered a new era – that of selectively blocking the single prostanoid receptor that is primarily responsible for pain and inflammation, thereby preserving the production and activity of homeostatic prostanoids (Nakao *et al*. 2007; Kawabata 2011).

This manuscript reviews the mechanism of action of anti‐inflammatory drugs for use in animals and introduce the first‐in‐class drug for managing pain and inflammation associated with osteoarthritis (OA) in veterinary patients: grapiprant, an EP4 prostaglandin receptor antagonist (PRA).

### AA cascade revisited

Arachidonic acid is an omega 6 polyunsaturated lipid that is an integral part of cell membranes and is liberated from the cell membrane by the enzyme phospholipase A_2_ (PLA_2_) as part of normal cellular metabolism and following cell injury (Curry *et al*. 2005; KuKanich *et al*. 2012). The freed AA molecule is then shuttled into one of three pathways that generate three groups of molecules: prostanoids, leukotrienes and epoxides (Curry *et al*. 2005; KuKanich *et al*. 2012). This manuscript will focus on the prostanoid group.

The enzymes cyclo‐oxygenase 1 and 2 (COX‐1 and COX‐2) convert AA into prostaglandin H_2_ (PGH_2_), and PGH_2_ is subsequently converted, via tissue‐specific isomerases and oxidoreductases, to five active prostanoid molecules: thromboxane (TXA), PGD_2_, PGI_2_ (prostacyclin), PGF_2*α*_ and PGE_2_ (Simmons *et al*. 2004; Ricciotti & FitzGerald 2011). COX‐1 is expressed constitutively in most cells and is the dominant source of prostanoids that are involved in gastric epithelial cytoprotection and homeostasis. COX‐2 is constitutively expressed in some tissues, but is also inducible by inflammatory stimuli, and is an important source of prostanoid formation in periods or locations of inflammation. In inflammation, the profile of prostanoid production is determined by the differential expression of these enzymes within cells at the site of inflammation (Ricciotti & FitzGerald 2011). Production of PGE_2_ and PGI_2_ predominates in sites where COX‐2 is activated (Simmons *et al*. 2004).

In the mid‐1990s, prostanoid receptors were identified and continue to be characterized (Woodward *et al*. 2011). These receptors have varying distributions throughout the body and mediate the activities of each prostanoid. Each prostanoid interacts with at least one distinct receptor to mediate specific physiological effects, and in some cases this includes specific receptor subtypes for a particular prostanoid.

### Inhibiting prostanoid production: the good and the bad

Prostanoids are responsible for a wide range of homeostatic functions in mammals including regulation of renal haemodynamics and ion transport, gastrointestinal cytoprotection and motility, vascular and bronchial smooth muscle activity, immune function and platelet aggregation (Woodward *et al*. 2011; KuKanich *et al*. 2012). Pain and inflammation are also mediated by prostanoids, in particular PGE_2_, and thus therapeutic drugs are often used to decrease the production and activity of this molecule (Curry *et al*. 2005; KuKanich *et al*. 2012). Steroids and cyclo‐oxygenase inhibiting drugs have until now been the primary options for treating pain and inflammation, and because of their mechanism of action, these approaches have some drawbacks that are discussed below (Stahn *et al*. 2007; KuKanich *et al*. 2012).

Corticosteroids inhibit the enzyme PLA_2_, acting up‐stream of the COX enzymes in inhibiting the metabolism of AA (Stahn *et al*. 2007). Corticosteroids prevent the synthesis of prostanoids, leukotrienes and epoxides, and thus have broad anti‐inflammatory action, but also inhibit the production of many homeostatic AA metabolites. Short‐acting corticosteroids can be important therapeutics in the management of acute allergic reactions, and while they may also provide some degree of pain relief, the untoward side effects make them a poor choice for long‐term treatment of pain in veterinary patients.

Salicylic acid is a natural product derived from plants such as willow bark and meadowsweet, and its medicinal properties of pain relief and fever reduction have been utilized for centuries (Vane 1971). Aspirin, or acetylsalicylic acid, is a synthetic drug made from salicylic acid. Aspirin irreversibly binds to the COX enzymes and inhibits the formation of all prostanoids (Flower 2003; KuKanich *et al*. 2012). While aspirin is effective in reducing fever, pain and inflammation, there are significant adverse effects of treatment with aspirin that are well‐recognized in humans and animals, in particular gastrointestinal irritation and ulceration. Furthermore, irreversible inhibition of platelet TXA by aspirin leads to reduced aggregation that lasts up to 7–10 days (the life span of the affected platelets). This anti‐thrombotic property can be useful in clinical conditions where reducing blood clotting is called for, but should also be recognized as a potentially significant unwanted side effect of aspirin administration.

Cyclo‐oxygenase inhibiting non‐steroidal anti‐inflammatory drugs (COX inhibiting NSAIDs) decrease inflammation through inhibition of COX enzymes and subsequent inhibition of prostaglanoid production (Curry *et al*. 2005; KuKanich *et al*. 2012). This class includes aspirin, as described above, as well as other non‐selective COX inhibitors (i.e. drugs that inhibit both COX 1 and COX 2 enzymes) such as ketoprofen and indomethacin. In addition, drugs have been developed that preferentially inhibit COX‐2, such as carprofen, meloxicam, deracoxib, firocoxib and robenacoxib.

The two cyclo‐oxygenase isoenzymes, COX‐1 and COX‐2, were isolated in the 1980s and shortly thereafter it was discovered that COX‐2 is induced in response to tissue injury and is associated with inflammation and pain (Vane 1971; KuKanich *et al*. 2012). Drug development then concentrated on molecules that preferentially inhibited this enzyme in the hope of sparing the so‐called ‘house‐keeping’ prostaglandins produced by COX‐1. The coxib class of drugs, or selective COX‐2 inhibitors, were first marketed in human medicine in 1999. In humans, COX‐2 selective products such as rofecoxib (Vioxx^®^ Merck & Co., Inc. Kenilworth, NJ USA) and celecoxib (Celebrex^®^ Pfizer Inc. New York, NY USA) did show improved GI tolerability; however, in some cases, the FDA reviewed evidence leading the agency to conclude that their use was related to significant cardiovascular side effects and thus, all but one product have been removed from the US market (Harirforoosh *et al*. 2013). The underlying cause for these adverse cardiovascular events may be attributed to inhibition of COX‐2 synthesis of PGI_2_ (prostacyclin). PGI_2_ is a potent vasodilator and has anti‐thrombotic properties (an endogenous antagonist of TXA), thus decreased production of PGI_2_ by coxib‐inhibiting NSAIDs may lead to increased thrombosis and vasoconstriction, two methods of potentiating cardiovascular disease (Harirforoosh *et al*. 2013). The cardiovascular adverse events associated with COX‐2 selective NSAIDs in humans have not been observed in dogs taking COX‐2 selective NSAIDs. However, severe gastrointestinal, renal and hepatic adverse events have been reported in dogs taking these products (Lascelles *et al*. 2005; KuKanich *et al*. 2012; Monteiro‐Steagall *et al*. 2013).

Both COX‐1 and COX‐2 activity are important in maintaining gastrointestinal integrity and renal haemodynamics in dogs, primarily through the actions of PGE_2_ and PGI_2_ (Wilson *et al*. 2004; KuKanich *et al*. 2012). COX‐2 is expressed in the canine kidney and involved in regulation of vessel tone and salt and water balance via synthesis of PGE_2_ and PGI_2_. In the stomach and intestinal tract, COX‐1 is the primary mediator of gastro‐protective prostanoid (PGE_2_ and PGI_2_) production in the healthy animal. COX‐2 is up‐regulated in areas of gastrointestinal irritation and it is important in promoting the healing of gastrointestinal ulcerations (KuKanich *et al*. 2012). This suggests that administration of a selective COX‐2 inhibitor drug in the face of gastrointestinal ulceration, such as due to stress or administration of corticosteroids or a non‐selective cyclo‐oxygenase‐inhibiting NSAID such as aspirin, may reduce healing and potentiate ulceration and perforation (Lascelles *et al*. 2005). Another potential mechanism for gastrointestinal injury secondary to cyclo‐oxygenase inhibiting NSAID administration is the increased production of inflammatory leukotrienes (Curry *et al*. 2005). Inhibition of the COX pathways may result in increased AA that must be metabolized by the lipo‐oxygenase enzyme (LOX) pathway.

The four FDA approved COX inhibitor NSAIDs for use in dogs, unless contra‐indicated, are considered to be effective treatments for the pain associated with OA. However, these COX‐inhibiting NSAIDs, as a class, carry the potential for adverse effects including gastrointestinal ulceration and perforation and renal insufficiency (Lascelles *et al*. 2005; Monteiro‐Steagall *et al*. 2013). While COX‐2 selective and preferential NSAIDs are associated with fewer side effects compared to aspirin and older, non‐selective COX inhibitor NSAIDs, many dogs do not tolerate these drugs, and alternative pharmacologic pain relief is indicated. Currently, no other class of pharmaceuticals is approved by the FDA for the treatment of pain and inflammation associated with OA in dogs. Thus, it is evident that an FDA approved product that demonstrates targeted pain relief with improved tolerability is needed.

## The piprant class: PRA

After the identification of prostanoid receptors in the 1990s, significant research ensued into methods of targeting each receptor with synthetic agonists and antagonists (Woodward *et al*. 2011). In October 2013, the World Health Organization defined a newly recognized class of drugs that act as PRAs as the piprant class (World Health Organization, 2013).

### EP4 PRAs and anti‐inflammatory effects: experimental models

Prostaglandin E_2_ (PGE_2_) is a key mediator of swelling, redness and pain, the classic signs of inflammation, with pain resulting from PGE_2_‐mediated sensitization of sensory neurons and swelling and redness resulting from PGE_2_‐mediated vasodilation and increased vascular permeability (Ricciotti & FitzGerald 2011). PGE_2_ exerts its effects via four receptors, EP1, EP2, EP3 and EP4 (Woodward *et al*. 2011). The EP4 receptor is the primary mediator of the PGE_2_‐elicited sensitization of sensory neurons and PGE_2_‐elicited inflammation (Southall & Vasko 2001; McCoy *et al*. 2002; Lin *et al*. 2006; Nakao *et al*. 2007; Clark *et al*. 2008; Chen *et al*. 2010; Boyd *et al*. 2011). Grapiprant (AT‐001) is a new analgesic and anti‐inflammatory drug in the piprant class that functions as a selective EP4 PRA (Nakao *et al*. 2007). The following is a review of the literature and studies that demonstrate the anti‐inflammatory effects of EP4 PRAs and of grapiprant in particular.

The use of receptor knockout mice has been helpful in understanding the roles of the specific EP receptors in inflammation. Collagen antibody‐induced arthritis (CAIA) in mice is a model for the human inflammatory disorder rheumatoid arthritis (RA). The individual PGE_2_ receptors were evaluated by inducing CAIA in mice which were genetically modified to knock out the EP1, ‐2, ‐3, or ‐4 receptors and measuring the clinical, histopathological and cellular markers of disease (McCoy *et al*. 2002). When mice with one of these four receptors knocked out were evaluated for signs of pain and inflammation, the EP4 receptor knock‐out mice, but not the EP1, EP2 or EP3 receptor knock‐out mice, had decreased incidence and severity of disease, decreased histopathological deterioration associated with arthritis and decreased levels of inflammatory markers, implicating EP4 as a key mediator of inflammation. In a mouse model of acute inflammation, EP1, 2, 3 or 4 receptor knock‐out mice were subjected to UV irradiation of the skin. EP4 and EP2 knock‐out mice, but not EP1 or EP3 knock‐out mice, had significantly decreased ear swelling compared to control mice. The reduction in swelling was also seen when the wild‐type mice were treated with an EP4 PRA (Kabashima *et al*. 2007).

The anti‐inflammatory effects of EP4 receptor antagonism have also been investigated in animal models of inflammation. In a study by Clark *et al*. (2008), joint inflammation (as measured by paw swelling) was created using an adjuvant‐induced arthritis (AIA) model in rats. Rats treated with an EP4 PRA (MF498), but not an EP1 or an EP3 PRA, demonstrated reduced paw swelling. The reduction in swelling was similar to that seen in mice treated with a cyclo‐oxygenase‐2 (COX‐2) inhibitor. In a study by Murase *et al*. (2008), the selective EP4 PRA, CJ‐042,794, caused decreased paw swelling in a complete Freund's adjuvant model of chronic inflammatory pain in rats. The reductions in paw swelling in the CJ‐042,794 treated rats were again similar to those seen with rofecoxib (a COX‐2 inhibitor) treated rats. Using a complete Freund's adjuvant administration as a rodent model for pain and inflammation, treatment with an EP4 PRA, identified as 1a, also demonstrated reduced swelling of the paw (Boyd *et al*. 2011). In a series of experiments reported by Chen *et al*. (2010), an EP4 PRA, ER‐819762, suppressed inflammatory cytokine production, suppressed disease and slowed disease progression in collagen and GPI‐induced arthritis in mice. These data support the anti‐inflammatory role of EP4 receptor antagonism in rodents. Indeed, these results demonstrated that EP4 PRAs can reduce inflammation as effectively as COX‐2 inhibitors.

Several studies have been conducted examining the anti‐inflammatory and analgesic effects of grapiprant (AT‐001, referred to as CJ‐023,423 in the publications) in rats (Nakao *et al*. 2007). Studies using rat models have demonstrated grapiprant's ability to reduce acute and chronic pain and inflammation (Nakao *et al*. 2007; RaQualia 2007a,b). The anti‐inflammatory effect of grapiprant on paw swelling, inflammatory biomarkers in the serum and synovial inflammation was examined in the tarsal joint in AIA in rats (RaQualia 2007b). Grapiprant exhibited dose‐dependent and significant anti‐inflammatory effects on all parameters tested. The efficacy of grapiprant on paw swelling was comparable to those of rofecoxib and piroxicam (COX‐inhibiting drugs). In another study, the anti‐inflammatory activity of grapiprant on rat carrageenan‐induced foot swelling was investigated (RaQualia 2007a). Grapiprant inhibited foot swelling in a dose‐dependent manner compared to rats treated with placebo control. Nakao *et al*. (2007) investigated three different models of pain in rats: mechanical hyperalgesia, thermal hyperalgesia and weight bearing deficit. Oral administration of CJ‐023,423 significantly reduced hyperalgesia in rodents in all three models, including significantly reversing complete Freund's adjuvant‐induced chronic inflammatory pain.

Not only is the EP4 receptor prominently involved in inflammation and pain, but importantly this receptor may mediate central sensitization and play a role in chronic pain. Lin *et al*. (2006) reported that in rodents, the EP4 receptor is expressed by sensory neurons, and the level of EP4 receptors increased following peripheral inflammation. Administration of an EP4 PRA decreased pain and hypersensitivity in rodents.

### EP4 PRA in veterinary medicine: targeted pain management

The EP4 receptor in mice, humans and dogs has been cloned and characterized. The canine EP4 receptor has approximately 90% homology to the human receptor (Castleberry *et al*. 2001). Grapiprant is a PRA that selectively blocks the EP4 receptor in rodents, humans, and dogs (RaQualia 2007c). Grapiprant has undergone experimental and pilot studies in laboratory and client‐owned dogs. A multi‐site, masked, placebo‐controlled, randomized field trial for the control of pain and inflammation associated with OA in dogs was recently completed and was submitted to and accepted by the FDA in support of regulatory approval (www.aratana.com). The results of these studies in client‐owned dogs demonstrate that this drug provides control of the pain associated with OA. Studies are currently underway investigating the use of grapiprant in cats, and at this time data are not yet available.

As part of development of grapiprant for FDA approval for dogs with OA, a safety study in healthy Beagles investigated the effects of daily orally administered doses up to 50 mg kg^−1^ day^−1^ oral suspension (equivalent to approximately 30.5 mg kg^−1^ day^−1^ of the tablet formulation) for nine consecutive months (Rausch‐Derra *et al*. 2015a,b). There were no drug‐related effects on liver enzyme values, BUN/creatinine, or platelet function. Mild and reversible dose‐ and time‐dependent decreases in total protein, albumin and calcium were seen. Clinical signs were also dose dependent and restricted to mild gastrointestinal signs including soft stool, occasional stool with mucus or blood, and sporadic vomiting. There were no histopathological changes to any tissues, including the stomach, kidneys or liver except in one dog in the 50 mg kg^−1^ group had mild mucosal regeneration of the ileum seen on histopathology.

The relative lack of toxic effects with grapiprant compared to those that have been reported in dogs treated with COX‐inhibiting NSAIDs is not surprising. Grapiprant is a targeted approach to pain management – it selectively blocks the EP4 receptor, does not interfere with the production of prostanoids, and therefore does not affect the other PG receptor pathways that are affected in animals treated with COX inhibitor NSAIDs.

## Conclusion

PGE_2_ is a key mediator of pain and inflammation and has its effect through binding to the EP4 receptor. Grapiprant directly and specifically blocks the EP4 receptor, and therefore blocks PGE_2_‐elicited pain and inflammation. An EP4 PRA, such as grapiprant, which has been demonstrated to provide relief from arthritic pain in canine clinical patients, acts without affecting the synthesis and widespread activity of PGE_2_ and other prostanoids.

Safety studies in laboratory dogs have demonstrated an excellent safety profile, and a wide safety margin, and this has been confirmed in two large field effectiveness studies in dogs with OA (Rausch‐Derra *et al*. 2015a,b). The relative lack of toxic effects with grapiprant compared to those that have been reported in dogs treated with COX‐inhibiting NSAIDs is consistent with grapiprant's unique mechanism of action, although until FDA review and approval, claims of safety and effectiveness cannot be made. Grapiprant may offer a more targeted, and potentially better tolerated, method of pain management in dogs with OA.

## Conflicts of interest

At the time of writing, all authors were employees of Aratana Therapeutics, Inc., Kansas City, KS, USA At the time of publication, KK Shaw will be a full time employee of Animal Surgical Clinic of Seattle; Seattle, WA, USA and a paid consultant for Aratana Therapeutics, Inc.
